# The Gut Microbial Lipid Metabolite 14(15)-EpETE Inhibits Substance P Release by Targeting GCG/PKA Signaling to Relieve Cisplatin-Induced Nausea and Vomiting in Rats

**DOI:** 10.4014/jmb.2403.03044

**Published:** 2024-07-15

**Authors:** Man Lu, Liwei Xie, Sijie Yin, Jing Zhou, Lingmei Yi, Ling Ye

**Affiliations:** 1 Department of Anesthesiology, The First Affiliated Hospital of Zhejiang Chinese Medical University (Zhejiang Provincial Hospital of Traditional Chinese Medicine), No. 54 Youdian Rd., Shangcheng District, Hangzhou, Zhejiang 310006, P.R. China; 2 Department of Anesthesiology, The First Affiliated Hospital, Zhejiang University School of Medicine, No. 79 Qingchun Rd., Hangzhou, Zhejiang 310006, P.R. China

**Keywords:** Chemotherapy-induced nausea and vomiting, gut microbial lipid metabolites, 14(15)-epoxyeicosatetraenoic acids, substance P, glucagon/protein kinase A signaling

## Abstract

Chemotherapy-induced nausea and vomiting (CINV) is a debilitating side effect related to activation of substance P (SP). SP activation can result from dysregulation of the gut-brain axis, and also from activation of protein kinase A signaling (PKA) signaling. In this study, we connected these factors in an attempt to unveil the mechanisms underlying CINV and develop new therapeutic strategies. Female rats were injected with cisplatin (Cis) to induce pica. Fecal samples were collected before/after injection, and subjected to lipid metabolomics analysis. In another portion of pica rats, the PKA inhibitor KT5720 was applied to investigate the involvement of PKA signaling in CINV, while fecal microbiota transplantation (FMT) was implemented to verify the therapeutic effect of the lipid metabolite 14(15)-EpETE. Pica symptoms were recorded, followed by ileal histological examination. The targeting relationship between 14(15)-EpETE and glucagon was determined by bioinformatics. SP and glucagon/PKA signaling in rat ileum, serum, and/or brain substantia nigra were detected by immunohistochemistry, enzyme-linked immunosorbent assay, and/or western blot. The results showed a significantly lower level of 14(15)-EpETE in rat feces after Cis injection. KT5720 treatment alleviated Cis-induced pica symptoms, ileal injury, SP content increase in the ileum, serum, and brain substantia nigra, and ileal PKA activation in rats. The ileal level of glucagon was elevated by Cis in rats. FMT exerted an effect similar to that of KT5720 treatment, relieving the Cis-induced changes, including ileal glucagon/PKA activation in rats. Our findings demonstrate that FMT restores 14(15)-EpETE production, which inhibits SP release by targeting GCG/PKA signaling, ultimately mitigating CINV.

## Introduction

Chemotherapy represents a mainstream type of cancer treatment, yet with side effects, of which chemotherapy-induced nausea and vomiting (CINV) is common. CINV can be so unbearable as to deter patients from accepting potentially curative chemotherapy [1]. Cisplatin (Cis) is a platinum-containing chemotherapeutic drug that has been widely applied for treatment of various cancers [2], through inducing adducts and crosslinks of DNA to impair cell division and cause apoptotic cell death [2, 3]. CINV occurs several days following Cis-based chemotherapy, bringing significant discomfort to patients and probably suggesting an unmet medical need [4]. Moreover, severe vomiting can seriously reduce quality of life and prolong the recovery time of postoperative intestinal function in hospitalized surgical patients [5]. Therefore, the use of vomiting model systems is urgently needed to elucidate their mechanisms of action and provide reference for better clinical application.

In response to chemotherapy, emesis is actually triggered as a defense mechanism mediated by the vomiting center (VC) of the brain [1, 5]. The VC integrates both the central and peripheral pathways, which are directly activated by the input in the area postrema (referred to as the chemoreceptor trigger zone (CTZ)) of the brain, and elicited by the transmission of the input from enterochromaffin cells in the gastrointestinal tract via the vagal afferents, upon chemotherapy, respectively [1, 5, 6]. The CTZ and the abdominal vagal afferents both contain various receptors for that input transduction, and include the receptor for substance P (SP); SP is a neurotransmitter of the tachykinin neuropeptide family and activates the Neurokinin-1 receptor (NK1R) as the final common pathway to induce an emetic response, or vomiting [1, 5, 6]. These highlight a bidirectional neural and endocrine communication between the brain and gastrointestinal tract, and suggest that chemotherapy modulates this gut-brain axis to cause CINV. Improvement of the gut microbiota and vagus nerve activity has been proven effective in relief of Cis-based CINV [7].

Arachidonic acid (ARA), a ω-6 polyunsaturated fatty acid (PUFA) essential to cellular inflammation and immunity [8], has been recognized as a potent emetogen along with some of its metabolites [9]. Moreover, its metabolism is closely pertinent to intestinal microecology [10]. Through performing ARA-targeting metabolomics analysis, significant downregulation of 14(15)-epoxyeicosatetraenoic acids (14(15)-EpETE) was identified in feces from Cis-induced pica rat models. 14(15)-EpETE is a lipid metabolite of eicosapentaenoic acid (EPA) [11], which is a ω-3 PUFA that uses the same series of enzymes during eicosanoid production as ARA, and competes with ARA for these enzymes to affect ARA metabolism [12]. Subsequently, the potential targets of 14(15)-EpETE were analyzed by the Swisstarget database, yielding glucagon (GCG). GCG activates protein kinase A (PKA) [6], a downstream regulator in the adenylyl cyclase (cAMP) signaling pathway, which has been established as playing an emetic role in cancer patients [13]. Moreover, PKA phosphorylation/activation positively correlates with the peak vomit frequency during both the acute and delayed phases of Cis-based CINV [6], and the release of SP and the activity of NK1R can be inhibited by PKA inhibitors [14, 15]. Therefore, we speculated that 14(15)-EpETE targets GCG to induce PKA signaling suppression, which inhibits the release of SP by decreasing the NK1R activity, eventually relieving Cis-based CINV.

Fecal microbiota transplantation (FMT) has been regarded as an effective treatment for chronic unpredictable mild stress (CUMS)-induced depression [16], the mechanism of which is the restoration of the gut microbiota of depressive rats to a healthy state [16]. Since 14(15)-EpETE is rare in feces of rats after Cis administration, we conjectured that FMT may ameliorate the gut microbiota of Cis-induced pica rats to increase the content of 14(15)-EpETE in the metabolites of their gut microorganisms, and thus relieve Cis-based CINV.

## Materials and Methods

### Establishment of Cis-Induced Pica Rats

Female Wistar rats (*n* = 51, aged 8 weeks, weighing 180 ± 20 g) were kept at a SPF laboratory animal facility under controlled conditions of 22 ± 1.0°C, 10% humidity, and a 12 h:12 h light/dark circadian cycle. Food and water were given to the rats ad libitum. All the rats were allowed to acclimatize for one week before use in experimentation.

To establish the pica rat models, Wistar rats were intraperitoneally injected with Cis (1134357, 6 mg/kg, Sigma-Aldrich, USA) [17]. ARA-targeting metabolomics analysis was performed to detect Cis-induced anomalies in the lipid metabolism of rats. Rat feces were collected from three Cis-injected rats at days 1, 2, and 3 before and after cisplatin injection, and used for ARA-targeting metabolomics analysis. Subsequent to the last feces collection, all these rats were intraperitoneally anesthetized with 1% pentobarbital sodium (P010, 150 mg/kg, Sigma-Aldrich), followed by euthanasia via cervical dislocation.

### ARA-Targeting Metabolomics Analysis through Liquid Chromatography-Tandem Mass Spectrometry (LC-MS/MS) and Identification of 14(15)-EpETE

Upon collection, rat feces (50 mg) were flash-frozen by liquid nitrogen, ground into powder, and then purified with 1 ml cold methanol, followed by vortexing for 5 min. The fecal sample was maintained in a 4°C refrigerator overnight. Later, 10 μl of 1 μM mixed internal standard was loaded. Centrifugation at 5,000 ×*g* was performed at 4°C for 10 min to obtain the supernate, which was dried under a stream of nitrogen, and then reconstituted in a 100 μl mixture of methanol and water at a volume ratio of 1:1. The obtained solution was again centrifuged for collecting a supernate, which was later used for LC-MS/MS analysis.

The supernate was transferred to an LC-ESI-MS/MS system (HPLC, Shim-pack UFLC SHIMADZU CBM30A, Shimadzu, Japan), and the MS, 6500 Q TRAP system (Applied Biosystems, USA) for metabolomics analysis conducted by Metware (China) [18].

### Grouping, KT5720 Treatment, and FMT Implementation

To investigate the effect of KT5720 on Cis-induced pica, rats were randomized into Con (control), Con+KT5720, Cis, and Cis+KT5720 groups (*n* = 6/group). Gavage with 1 mM KT5720 at a dose of 5 ml/kg was conducted with rats once, one day before sampling (Con+KT5720) or before Cis injection (Cis+KT5720) [19]. Dimethyl sulfoxide (34869, Sigma-Aldrich) was applied in the same manner as the control of KT5720 to the Con and Cis groups.

Thereafter, the therapeutic effect of FMT on Cis-induced pica was determined through experiments conducted by setting up Con, Con+FMT, Cis, and Cis+FMT groups, with 6 rats randomly assigned into each group. Transplantation with fecal bacterial fluid of the rats from the Con group via gavage (1 ml/100 g body weight) was implemented once a week, for a total of three weeks, with healthy rats (Con+FMT) or with Cis-injected rats (Cis+FMT) after Cis injection [16], whereas rats in the Con and Cis groups received gavage with normal saline (1 ml/100 g body weight) in the same manner.

Normal saline was given to the rats as the control of Cis at the same amount in both Con groups as in the above two experimental modules. Laboratory rats seem to lack an emetic response, and eating nonnutritive substances such as Kaolin is analogous to having an emetic reflex, so it is therefore considered an alternative for nausea and vomiting responses [20]. The intake of Kaolin and food was measured and the body mass was recorded at 0, 24, 48, 72, and 96 h post Cis injection. Four weeks after Cis injection, under anesthetization with 1% pentobarbital sodium, all the rats were subjected to blood collection through the angular vein, and then sacrificed, as aforementioned. After rat sacrifice, the ilea and brain substantia nigra were collected.

### Hematoxylin-Eosin (H&E) Staining

Rat ilea were fixed in 4% paraformaldehyde (P885233, MACKLIN, China) for 24 h, and treated with gradient ethanol for dehydration, followed by transplantation using xylene. The ilea were then immersed in paraffin (1496904, Sigma-Aldrich), and sliced to a thickness of 4 μm. After being dewaxed using xylene, the slices were rehydrated with gradient ethanol. Hematoxylin (HY-N0116, MedChemExpress, USA) was subsequently used to stain the slices for 10 min. Next, the slices were distinguished with 1% hydrochloric ethanol, and rinsed by weakly alkaline water for developing blue, following which eosin (HY-D0505A, MedChemExpress, USA) was added for 1 min. Later, the slices were dehydrated, transparentized, and sealed in neutral balsam (N861409, Macklin, China). Through an optical microscope (ZEISS Primotech, Carl Zeiss, Germany), pathological changes in the ilea were observed under × 40 magnification.

### Immunohistochemistry

The ileal slices were dewaxed, dehydrated, and immersed in 3% H_2_O_2_ for quenching endogenous peroxidase activities, after which antigen retrieval was conducted by microwaving the slices in 0.01 mol/l citrate buffer (C9999, Sigma-Aldrich) for 4 min. The slices were then blocked using 5% goat serum (16210072, Thermo Fisher, USA) in 0.3% Triton X-100 (HFH10, Thermo Fisher, USA)-added phosphate-buffered saline (PBS; HY-K3005, MedChemExpress) at 37°C for 5 min, and incubated with antibody for SP (ab14184, Abcam, UK) at 4°C overnight. Later, the slices were washed thrice with PBS, and probed for 1 h by secondary antibody HRP-conjugated goat anti-mouse IgG (ab205719, Abcam,), followed by antibody visualization in DAB substrate (D8001, Sigma-Aldrich). Hematoxylin (H9627, Sigma-Aldrich) was added for nuclear counterstaining. The slices underwent the routine procedure described in H&E staining before being subjected to the observation of SP-positive areas by the optical microscope under × 100 magnification.

### Enzyme-Linked Immunosorbent Assay (ELISA)

Rat blood was settled on ice for 30 min and centrifuged at 2,000 ×*g* for 20 min at 4°C for harvest of serum. Rat brain substantiae nigrae were homogenized in PBS to obtain homogenates that were then centrifuged at 5,000 ×*g* for 5 min for supernate collection. An ELISA kit (CSB-E08358r, CUSABIO, China) was utilized to determine the level of SP in the serum and supernate. Briefly, 100 μl of the sample was added into enzyme-labeled plates, followed by the addition of 100 μl Biotin-antibody for 1-h incubation at 37°C, and later each well was washed with wash buffer. The liquid was removed, and after 1-h incubation with 100 μl HRP-avidin at 37°C in the plate, 90 μl TMB substrate was applied for color development. The reaction was terminated by 50 μl stop solution, and the measurement of the absorbance at 450 nm was completed by a microplate reader (iMark, Bio-Rad Laboratories, Inc., USA).

### Western Blot

Rat ilea were lysed in ice-cold PMSF (36978, Thermo Fisher)-added RIPA Buffer (89900, Thermo Fisher) for total protein obtainment. The protein was quantified by a BCA kit (CW0014, cwbiotech, China). Next, 20 μg of the protein was subjected to electrophoresis on SDS-PAGE gel (20328ES50, Yeasen, China), and then transferred onto a PVDF membrane (P2438, Sigma-Aldrich). Following being blocked in 5% nonfat dry milk at room temperature for 1 h, the membrane was incubated at 4°C overnight with antibodies for GCG (ab92517, 21 kDa, 0.3 μg/ml, Abcam), PKA (4782, 42 kDa, 1:1000, Abcam, Cell Signaling Technology, USA), and GAPDH (ab8245, 37 kDa, 1:10000, Abcam). The membrane was washed, followed by incubation with goat anti-rabbit IgG/Goat anti-Mouse IgG (A32731/31460, Thermo Fisher) at room temperature for 2 h. ECL substrates (1705061, Bio-Rad, USA) were added for visualization of protein bands, and the band quantification was conducted using Gel Imagestem (version 4.0, Tanon, China).

### Statistical Analysis

GraphPad prism (version 8.0, GraphPad Software Inc., USA) was utilized for data analysis, and measurement data were represented as mean ± SD. Comparisons between two groups of data were performed with independent *t*-test. One-way analysis of variance was adopted to compare the means of multiple groups, except for those of the groups in Figs. 3A-3C and 4A-4C, which were compared using two-way analysis of variance. Differences were considered statistically significant, when *p* < 0.05.

## Results

### 14(15)-EpETE Was Identified and Showed a Lowered Level in the Feces of Rats after Cis Injection

**Fig. 1:**
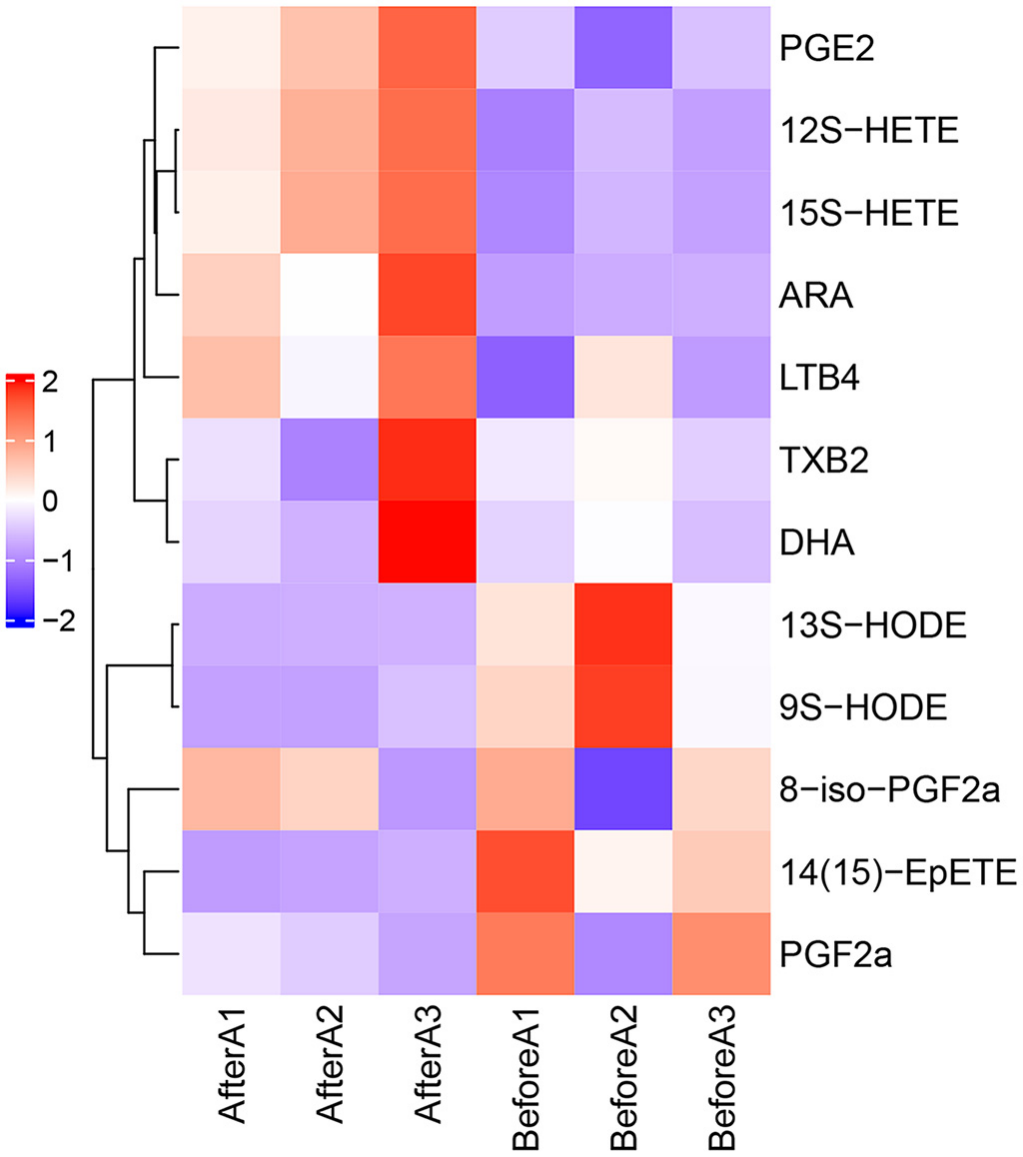
Hindrance of 14(15)-EpETE and boost of ARA and its metabolites by Cis injection in feces of rats. A heatmap drawn based on ARA-targeting metabolomics analysis of feces obtained beforeA1/2/3 and afterA1/2/3 from rats intravenously injected with 6 mg/kg Cis. *n* = 3. (ARA, arachidonic acid; 14(15)-EpETE, 14(15)-epoxyeicosatetraenoic acids; Cis, cisplatin; AfterA1/2/3, 1/2/3 days after Cis injection; BeforeA1/2/3, 1/2/3 days before Cis injection)

**Fig. 2:**
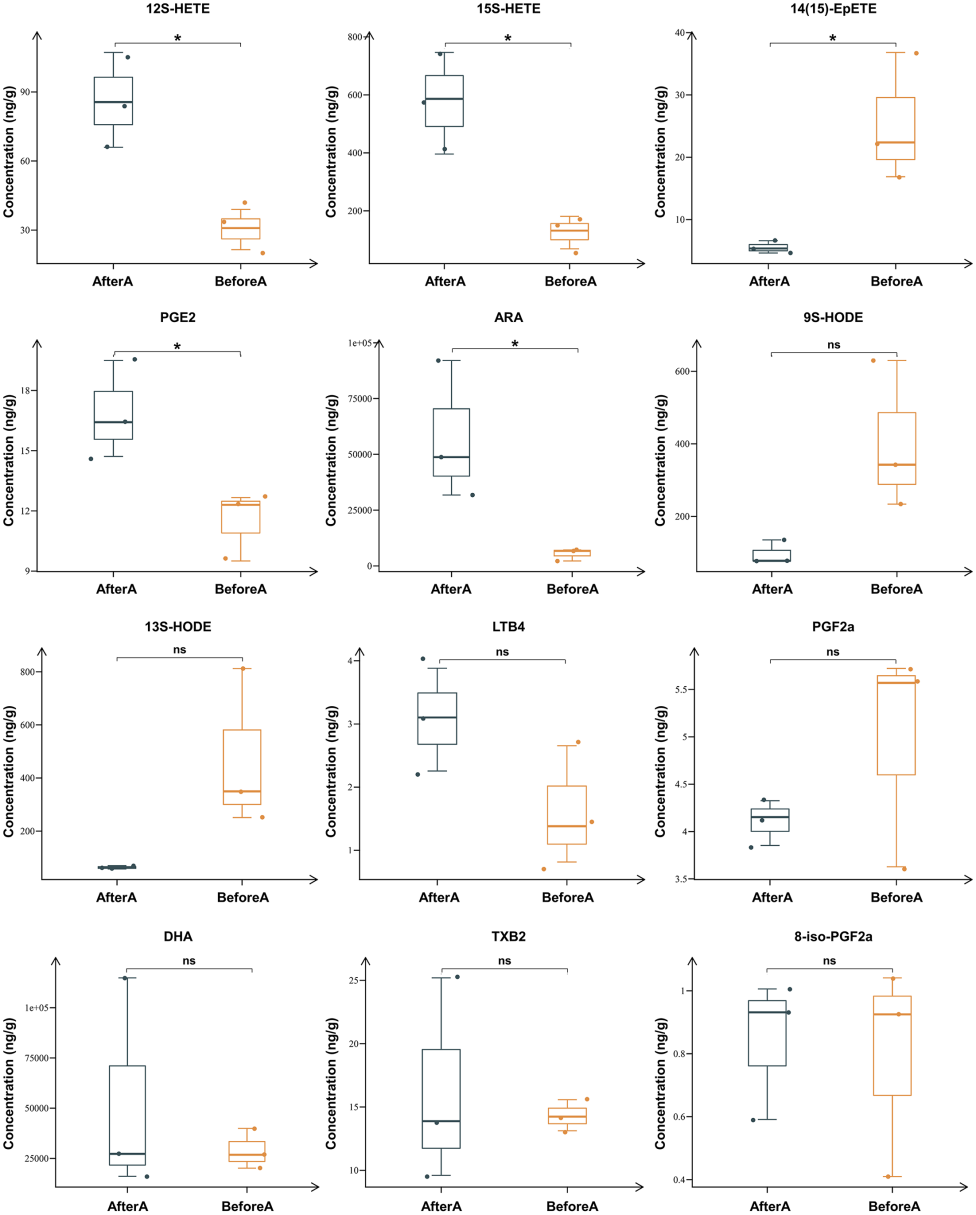
Lower level of 14(15)-EpETE in feces of rats after Cis injection. The quantitative results of 14(15)-EpETE, as well as PUFAs and their metabolites in feces obtained from rats three days before and after injection with 6 mg/kg Cis. *n* = 3, *p < 0.05 (14(15)-EpETE, 14(15)-epoxyeicosatetraenoic acids; Cis, cisplatin; PUFAs, polyunsaturated fatty acids; ns, no significance)

A heatmap was drawn based on ARA-targeting metabolomics analysis of feces obtained from rats intravenously injected with Cis. The results at days 1, 2, and 3 post Cis injection (Fig. 1) are represented by different colors, with deep red indicating a significant upregulation of the component, and deep blue indicating a significant downregulation. In Fig. 2, the presence or absence of significance symbols indicated the presence or absence of significant differences before and after Cis injection. The results in Figs. 1 and 2 show increased levels of ARA and its metabolites (12S-HETE, 15S-HETE, and PGE2), which were detected in the feces of the rats, while the contents of 14(15)-EpETE were found to be significantly reduced (*p* < 0.05, Figs. 1, 2). The contents of 9S-HODE, 13S-HODE, LTB4, PGF2a, DHA, TXB2, and 8-iso-PGF2a were barely changed in feces of rats before and after Cis injection (Figs. 1 and 2).

### Inactivation of the GCG/PKA Pathway Led to Anti-CINV Outcomes with Reduced Ileal Injury and SP Release in Cis-Induced Pica Rats

**Fig. 3:**
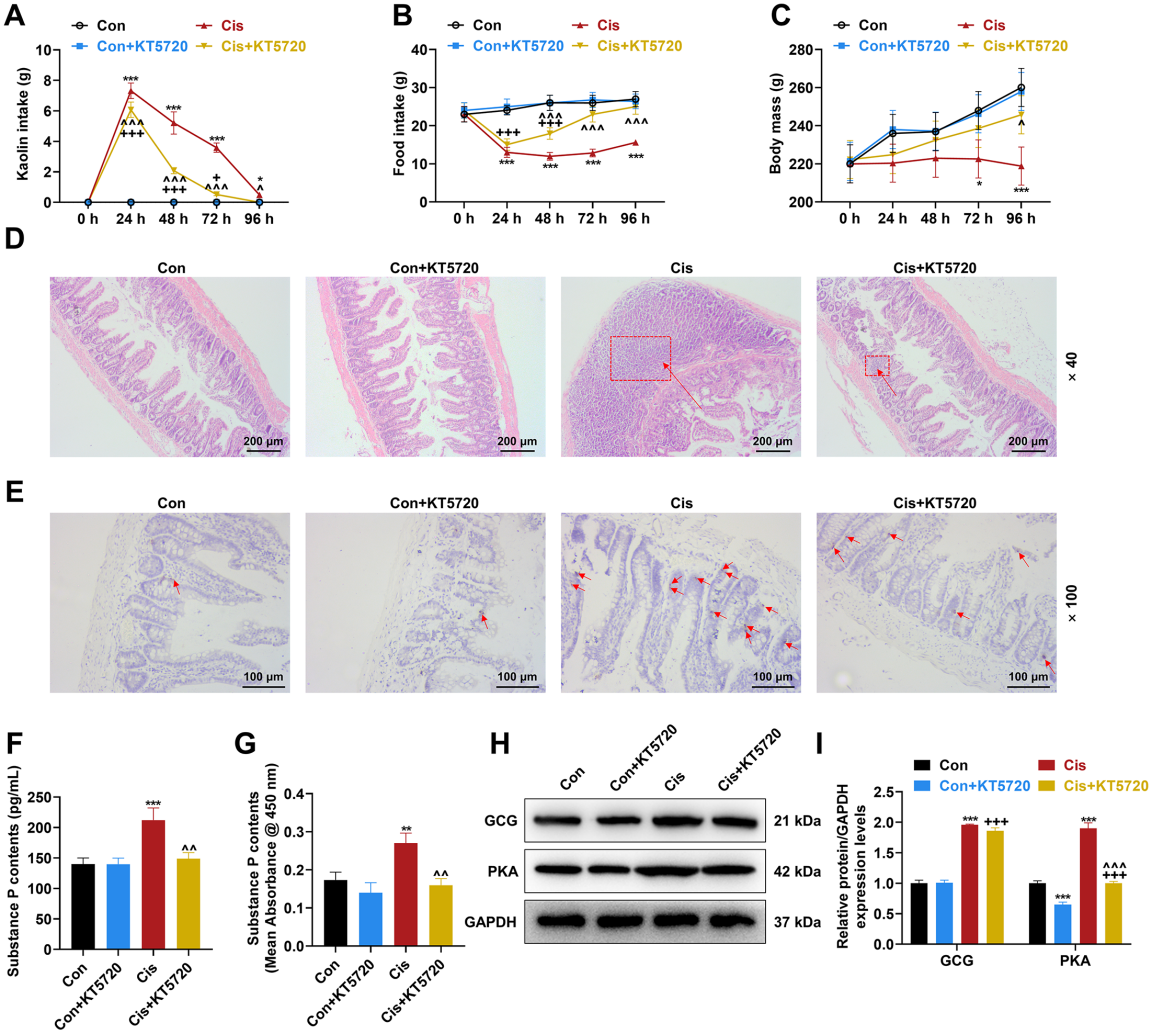
The relationship between inactivation of the GCG/PKA pathway and anti-CINV outcomes with reduced ileal injury and SP release in Cis-induced pica rats. (**A-I**). Rats underwent gavage with 1 mM KT5720 at a dose of 5 ml/kg or with an equal volume of dimethyl sulfoxide once, one day before Cis (6 mg/kg) injection. (**A-C**). The intake of Kaolin and food was measured and the body mass was recorded at 0, 24, 48, 72, and 96 h post Cis injection. (**D**). The histological abnormalities in the rat ileum were observed through hematoxylin-eosin staining (magnification, × 40; scale bar, 200 μm), and the inflammatory cells were indicated with arrows. (**E**). The SP-positive cells in the rat ileum were detected by immunohistochemistry (magnification, × 100; scale bar, 100 μm). (**F/G**). The content of SP in the serum and brain substantia nigra of rats was determined by enzyme-linked immunosorbent assay. (**H/I**). The expressions of GCG and PKA in the rat ileum were analyzed by western blot, with GAPDH serving as the internal control gene. *n* = 6 rats/group, *p < 0.05, **p < 0.01, ***p < 0.001, *vs. Con; ^*p* < 0.05, ^^*p* < 0.01, ^^^*p* < 0.001, ^vs. Cis; ^+^*p* < 0.05, ^+++^*p* < 0.001, ^+^vs. Con+KT5720 (Con, control; Cis, cisplatin; SP, substance; GCG, glucagon; PKA, protein kinase A; CINV, chemotherapy-induced nausea and vomiting).

To examine the involvement of protein kinase A (PKA) in Cis-induced chemotherapy-induced nausea and vomiting (CINV), rats were administered the PKA inhibitor KT5720 intragastrically one day before Cis injection, and subsequent pica symptoms were recorded. As shown in Fig. 3A-3C, Cis injection augmented the intake of Kaolin (*p* < 0.05, Fig. 3A) and reduced food intake (*p* < 0.001, Fig. 3B), 24, 48, 72, and 96 h after the injection, while decreasing the body mass of rats at post-injection 72 and 96 h (*p* < 0.05, Fig. 3C); however, these effects of Cis injection were in part offset by KT5720 gavage (*p* < 0.05, Fig. 3A-3C). In rats receiving KT5720 gavage, Cis injection exerted a similar effect (*p* < 0.05, Fig. 3A-3C), increasing Kaolin intake (Fig. 3A) but decreasing food intake (Fig. 3B) and body mass (Fig. 3C). H&E staining was then performed to gauge the pathological condition of the ilea of rats undergoing KT5720 gavage and/or Cis injection. The results showed that rats with Cis injection presented irregularly arranged ileal villi, significantly widened subepithelial space, and epithelial cell loss, accompanied by infiltration of a large number of inflammatory cells (Fig. 3D). Despite no obvious impact on the condition of the ileum, KT5720 gavage alleviated the above Cis injection-caused pathological conditions of the rat ilea (Fig. 3D). Furthermore, through immunohistochemistry, SP-positive cells were observed to exist in higher numbers in the ilea of rats with Cis injection compared to the control rat ilea (Fig. 3E), whereas KT5720 gavage resisted the Cis-induced increase in the number of SP-positive cells, though it did not affect the number of SP-positive cells in the control rat ilea (Fig. 3E). The ELISA results also revealed an elevated level of SP in the serum and in the brain substantiae nigrae of rats after Cis injection (*p* < 0.01, Fig. 3F-3G), and this elevation of the SP level was attenuated by KT5720 gavage (*p* < 0.01, Fig. 3F-3G). In addition, Cis injection boosted the expressions of GCG and PKA in the ilea of control rats (*p* < 0.001, Fig. 3H-3I). KT5720 gavage generated no impact upon the GCG expression, while inhibiting the expression of PKA in the control rat ilea (*p* < 0.001, Fig. 3H-3I). Moreover it mitigated the effect of Cis injection on the expression of PKA in the ileum (*p* < 0.001, Fig. 3H-3I). Cis injection increased the expression of GCG in the ilea of rats receiving KT5720 gavage, and reversed KT5720 gavage-induced PKA downregulation in the rat ilea (*p* < 0.001, Fig. 3H-3I).

### FMT Exerted an Anti-CINV Effect with Reduced Ileal Injury and SP Release and Blocked GCG/PKA Pathway in Cis-Induced Pica Rats

**Fig. 4:**
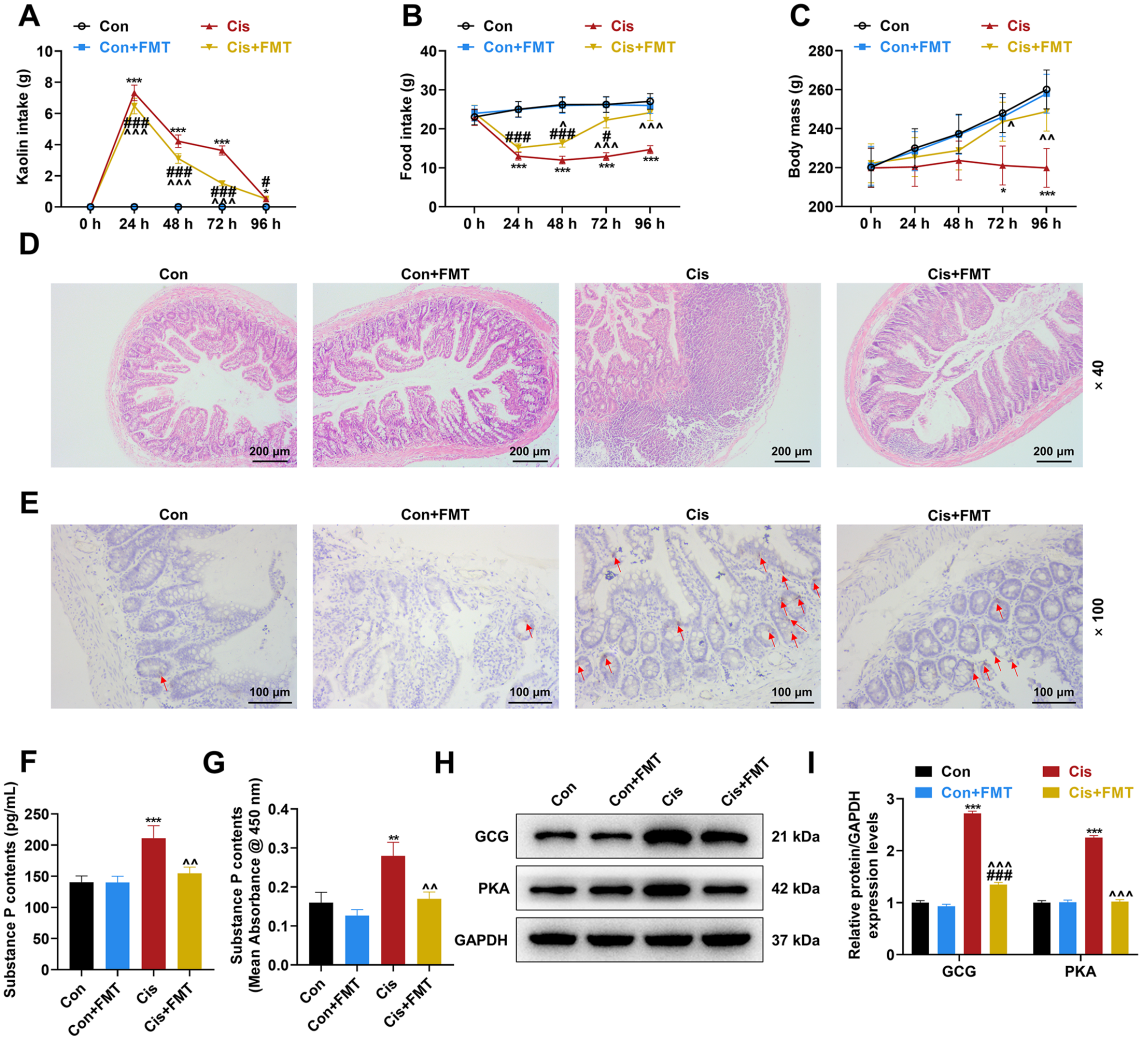
The effect of FMT on reduced ileal injury and SP release and blocked GCG/PKA pathway in Cisinduced pica rats. (**A-I**). Rats were injected with 6 mg/kg Cis, and then implanted with fecal bacterial fluid (1 ml/100 g body weight) of the rats from the Con group or with (1 ml/100 g body weight) normal saline via gavage, once a week, for a total of three weeks. (**A-C**). The intake of Kaolin and food was measured and the body mass was recorded at 0, 24, 48, 72, and 96 h post Cis injection. (**D**). The histological abnormalities in the rat ileum were observed through hematoxylin-eosin staining (magnification, × 40; scale bar, 200 μm). (**E**). The SP-positive cells in the rat ileum were detected by immunohistochemistry (magnification, × 100; scale bar, 100 μm). (**F, G**). The content of SP in the rat serum and brain substantia nigra was determined by enzyme-linked immunosorbent assay. (**H, I**). The expressions of GCG and PKA in in the rat ileum were analyzed by western blot, with GAPDH serving as the internal control gene. *n* = 6 rats/group, *p < 0.05, **p < 0.01, ***p < 0.001, *vs. Con; ^*p* < 0.05, ^^*p* < 0.01, ^^^*p* < 0.001, ^vs. Cis; ^#^*p* < 0.05, ^###^*p* < 0.001, ^#^vs. Con+FMT (FMT, fecal microbiota transplantation; Con, control; Cis, cisplatin; SP, substance; GCG, glucagon; PKA, protein kinase A; CINV, chemotherapy-induced nausea and vomiting).

FMT exhibits a therapeutic effect on depression [16], which prompts us to conjecture that FMT may relieve Cis-induced nausea and vomiting. To test this conjecture, FMT was given to rats once a week for three weeks after Cis injection. FMT exerted no obvious effect on the intake of Kaolin and food and the body mass of the control rats, but partly abrogated Cis injection-caused augmentation of the intake of Kaolin and reduction of the food intake and body mass of rats (*p* < 0.05, Fig. 4A-4C). The detrimental effect of Cis injection was also detected in rats receiving FMT gavage (*p* < 0.05, Fig. 4A-4C). Moreover, as presented through H&E staining, Cis injection-caused pathological conditions in the rat ilea were ameliorated by FMT; however, implementation of FMT alone did not impact on the ileal histology of the control rats (Fig. 4D). Meanwhile, the content of SP in the ileum, serum and brain substantia nigra of the control rats was not conspicuously altered after FMT implementation; yet, FMT attenuated the upregulation of SP by Cis injection in these three sites (Fig. 4E; *p* < 0.01, Fig. 4F-4G). Of note, the Cis injection-caused promotion of the GCG and PKA expressions in the rat ilea was attenuated by FMT (*p* < 0.001, Fig. 4H-4I), while FMT exerted no impact on the GCG and PKA expressions in the control rat ilea. Cis injection only apparently upregulated GCG in the ilea of rats treated with FMT (*p* < 0.001, Fig. 4H-4I).

## Discussion

CINV frequently develops following chemotherapy for cancer treatment [4]. Meanwhile, the gut microbiota affects lipid metabolism [21], and its dysbiosis serves as an important incentive to the chemotherapy-induced gastrointestinal toxicity that causes nausea and vomiting [22]. Cisplatin (Cis), an anti-cancer chemotherapeutic drug, can be highly emetogenic [4]. In the current study, we identified that the lipid metabolite 14(15)-EpETE produced content variations after Cis injection, and implemented FMT with Cis-injected pica rats to discover that improving the composition of gut microbiota relieved Cis-induced pica and intestinal injury by suppressing the GCG/PKA pathway. Since GCG is a target of 14(15)-EpETE based on our above findings, we inferred that FMT may ameliorate lipid metabolism to increase the content of 14(15)-EpETE, which restrains the GCG/PKA pathway, thus relieving Cis-based CINV.

In addition, 14(15)-EpETE is a metabolite of EPA [11], which is a representative ω-3 PUFA [12]. PUFAs can be metabolized through three metabolic (cyclooxygenase (COX), lipoxygenase (LOX), cytochrome p450 (CYP450)) pathways into a myriad of eicosanoids/oxylipins, bioactive mediators [11]. Epoxy-metabolites like 14(15)-EpETE are generated from the oxidation of EPA through the CYP450 pathway [11]. EPA and other ω-3 PUFAs exert an anti-inflammatory effect by producing anti-inflammatory bioactive compounds, and share the above oxylipin-synthesizing pathways with ω-6 PUFAs [11, 23, 24], such as ARA, which is released upon inflammatory reaction and induces proinflammatory eicosanoid mediators [25]. Moreover, they can compete with ω-6 PUFAs for these pathways, which may lead to altered metabolic profiles of ARA [12], and thus impinge on the state of inflammation of tissues, including the gut [24]. Furthermore, EPA enrichment co-occurs with a decrease in the content of ARA [26]. CINV is accompanied by inflammation of jejunum tissues [17]. Through ARA-targeting metabolomics analysis, we found that the content of 14(15)-EpETE was decreased, while the contents of ARA and the levels of ARA metabolites 12S-HETE, 15S-HETE and PGE2 were increased in feces of rats after Cis injection, indicating that ARA content was increased, with its metabolism outweighing that of PHA in Cis-based CINV. Also, 14(15)-EpETE may be protective against CINV as it is a metabolite of the anti-inflammatory EPA.

Bioinformatics analysis further predicts that 14(15)-EpETE targets GCG, which is a peptide hormone with pleiotropic effects, including hyperglycemia, decreased food intake, stimulation of lipolysis, and inhibited lipid synthesis [27]. GCG can activate PKA signaling by increasing the intracellular cAMP level in insulin-resistant animal models [28]. PKA is a cAMP-dependent protein kinase, and its activation by increased intracellular cAMP level further induces activation of Cav2.2 channels (N type), NMDA receptors, and the mammalian target of rapamycin, all of which contribute to the release of SP [15]. This might well explain the emetic role of PKA in dogs and human [29, 30], as SP is a neurotransmitter expressed by enterochromaffin cells in the peripheral nervous system and sensory nerve endings in the central nervous system upon chemical stimulation, as in chemotherapy, further resulting in NK1R-mediated emetic response, or vomiting [1, 5, 6]. Using the PKA inhibitor EPA, we confirmed that targeting the GCG/PKA pathway reduced the intake of Kaolin, a proxy for emesis in rats [20], while augmenting the food intake and body mass in Cis-induced pica rats. Moreover, the occurrence of CINV is associated with gut-brain axis alteration induced by disorganized pathways involving gut inflammation and disruption of the gut microbiome [31], and we found inhibition of the GCG/PKA pathway can ameliorate ileal inflammation and histological abnormalities in Cis-induced pica rats. Moreover, SP is secreted both in the gut and brain for induction of CINV and circulated through blood [6]. Our work showed that the GCG/PKA pathway inhibition caused downregulation of SP in the ileum, brain substantia nigra, and serum of Cis-induced pica rats. Taken together, our results suggested that 14(15)-EpETE targeted the GCG/PKA pathway to relieve Cis-induced CINV, with assistance from downregulation of SP.

The gut microbiota regulates the gut-brain axis to impact on CINV [7]. When being supplemented with *Clostridium butyricum*, the antiemetic agent thalidomide exerts a more potent CINV-ameliorating effect [7]. FMT restores the composition of the gut microbiota of depressive rats to a healthy state, which is considered as a therapeutic approach to depression [16]. Given that the microbiota interacts with ω-3 PUFAs to modulate the gut immune system [32], FMT may improve EPA metabolism in Cis-induced pica rats to a normal level with enhanced production of 14(15)-EpETE, which further acts to relieve CINV by targeting the GCG/PKA pathway, which was later verified as reliable by our study. However, the reliability may be challenged, as we did not detect and compare the content of 14(15)-EpETE in Cis-induced pica rats before and after FMT.

In conclusion, the current study demonstrated using in vivo experiments that the content of 14(15)-EpETE is decreased significantly in feces of rats after Cis injection, and FMT may enhance 14(15)-EpETE production to relieve CINV by targeting the GCG/PKA pathway. These findings illuminate the lipid metabolism-related mechanism underlying CINV, and provide insight for therapeutic strategies against it.
